# Effects of Subsurface Drip Irrigation Depth on Growth Characteristics and Yield Quality of Apples (*Malus pumila* Mill.) in Northwest China

**DOI:** 10.3390/plants14172702

**Published:** 2025-08-29

**Authors:** Ming Zheng, Yan Sun, Weiyi Mu, Yungang Bai, Quanjiu Wang, Zhenlin Lu, Wantong Zhang

**Affiliations:** 1 Institute of Water Resources and Hydropower, Xi’an University of Technology, Xi’an 710048, China; xjzhengming@126.com (M.Z.); 11414014@zju.edu.cn (Y.S.); weiyimu@xaut.edu.cn (W.M.); 2 Xinjiang Institute of Water Resources and Hydropower Research, Urumqi 830049, China; xjluzhenlin@126.com; 3 College of Resources and Environment, Xinjiang Agricultural University, Urumqi 830052, China; zhangwantong116@163.com

**Keywords:** nutrients, endogenous hormones, photosynthesis, SPAD, plant water potential

## Abstract

Subsurface drip irrigation can improve crop water and fertilizer use efficiency, but it can cause soil hypoxia. We report on experiments performed in Aksu Prefecture, Xinjiang (41°17′ N latitude, 80°17′ E longitude), from April 2023 to October 2024 using oxygenated drip irrigation from the surface to 50 cm depth in an apple (*Malus pumila* Mill.) orchard, to examine the effects of drip irrigation on inter-root hypoxia, tree growth, fruit quality, and yield. Compared with surface oxygenated drip irrigation (CK), irrigating at 10 and 30 cm increased soil water content in the root system, elevated gibberellin, zeatin ribosides, and indoleacetic acid contents and reduced abscisic acid contents in new shoot tips. Compared with CK, branch and leaf nitrogen, phosphorus, and potassium contents were increased with irrigation at depths of 10 and 30 cm. The leaf nitrogen (N), phosphorus (P), and potassium (K) contents were increased by 18.03%, 22.42%, and 16.63%, respectively, in the treatment with a burial depth of 30 cm. Among treatments, irrigation at 30 cm produced the highest average daily plant water potential, and irrigation at 50 cm was the lowest. Maximum leaf soil–plant analysis development (SPAD) values occurred when irrigated at 30 cm, and minimum values occurred at 50 cm. For both years, the largest range of light flux utilization occurred when irrigated at 30 cm and the lowest when irrigated at 50 cm. Significant correlations between indoleacetic acid (IAA), total gibberellin (GA), zeatin riboside (ZRs), leaf N content, leaf K content, plant water potential (PWP), net photosynthetic rate (Pn), SPAD, and apple yield were determined by partial mantel analysis. A significant correlation was found between abscisic acid (ABA), IAA, GA, leaf P and K content, and apple quality. Principal component analysis revealed a burial depth of 30 cm had the highest principal component composite score, indicating that this burial depth, and oxygenation and fertilization regime most favored apple growth, yield, and quality.

## 1. Introduction

Water resources limit agricultural production, and this affects sustainability and economic development [1]. Increasing demand for limited water resources necessitates improved irrigation management and techniques. Surface drip irrigation can more effectively conserve water resources than conventional surface irrigation, but soil evaporation is high, crop water-use efficiency is low, and rapid weed growth can occur. It is difficult to remove weeds without damaging the irrigation system except for suspended drip irrigation systems, because weeds and their roots entangle or otherwise block the irrigation belt drip head, and mowers can sever the irrigation belt itself. More practical and efficient, water-saving drip irrigation technologies for orchards is required.

Subsurface drip irrigation not only reduces the amount of ineffective evaporation from surface soils, but also delivers water and nutrients directly to the crop roots. This more effectively provides water and nutrients to the crop root system, reduces fertilizer loss and does not promote weed growth, enhances crop water use efficiency [2], and improves fruit quality and yield [3]. Such subsurface devices are usually buried at depths of 10–40 cm, depending on the crop [4,5,6]. However, drip irrigation can produce a wetting front, resulting in the forced expulsion of soil gases [7]. High-frequency drip irrigation scenarios in particular may lead to prolonged inter-root oxygen deprivation in crops [8,9], especially in clay soils [10]. This affects, among other things, soil fertility [11], and causes stomatal closure and reduces plant water potential, which can affect a crop’s normal physiological functions [12,13]. The plant then reduces water and nutrient uptake and transportation to above-ground parts, reducing crop water and nutrient utilization efficiency, and decreasing crop biomass [14]. This is a major limiting condition for high-crop yields.

Oxygenated irrigation is a new technology that increases dissolved oxygen concentrations in irrigation water through a water–fertilizer–air integration system. The water and air mixture is delivered to the soil in the crop’s root zone through drip irrigation pipes [15]. This technology is low cost and simple to operate, especially when irrigation and air injection are performed through the same drip irrigation pipe-line. This can promote crop growth and resolve the problem of reduced loam and clay loam soil oxygen content caused by short-term high irrigation saturation. Soil oxygen enables plant root to metabolize normally [16], relieves the plant of hypoxic stress, improves the root system structure to increase the plant water supply, and thus increases leaf photosynthesis rates [17]. However, with increased burial depth of the drip irrigation system, oxygen diffusion from the inter-root wetting front decreases and the crop remains potentially hypoxic [18]. No detailed studies on the effects of oxygenated irrigation and subsurface drip irrigation depth on the physiology and growth of fruit trees exist, especially under different irrigation conditions.

For apples (*Malus pumila* Mill.), we (1) study the effects of different drip irrigation depths + oxygenated drip irrigation on plant endogenous hormones; (2) analyze the effects of drip irrigation depth + oxygenated drip irrigation on soil–plant analysis development (SPAD), plant water potential, and photosynthetic characteristics, and analyze the correlation between physiological indicators and yield and quality; (3) explore the optimal drip irrigation depth for maximum yield and quality scores using principal component analysis.

## 2. Materials and Methods

### 2.1. Overview of the Experimental Area

**Figure 1:**
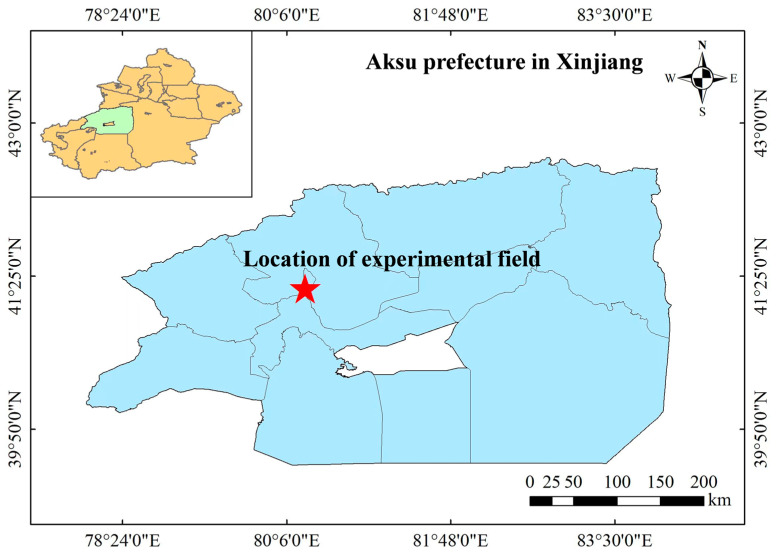
Location of experimental field.

Experiments were performed from April 2023 to October 2024 in a field at the Experimental Base of Specialty Forest Fruit of Xinjiang Agricultural University, Hongqipo Farm, Aksu Region, Xinjiang, China (41°17′ N latitude, 80°17′ E longitude, Figure 1). The test site has a continental temperate arid climate, according to local meteorological data statistics, with the last ten years averages for temperature (11.2 °C), effective cumulative temperature (3950 °C), average annual rainfall over the last ten years (about 74.4 mm), evaporation (about 2100 mm), total solar radiation (544.11–590.16 kJ cm^−2^), sunshine (2855–2967 h), and frost-free period (205–219 d). Soil average bulk weight is 1.51 g cm^−3^, soil texture is powdery loam, the field capacity of soil saturation a is 34.67%, the permanent wilting point is 15.42%. The clay, silt, and sand of soil content are 18.07%, 61.49%, and 20.45%, respectively. The water table is at 37 m.

### 2.2. Experimental Characteristics

**Figure 2:**
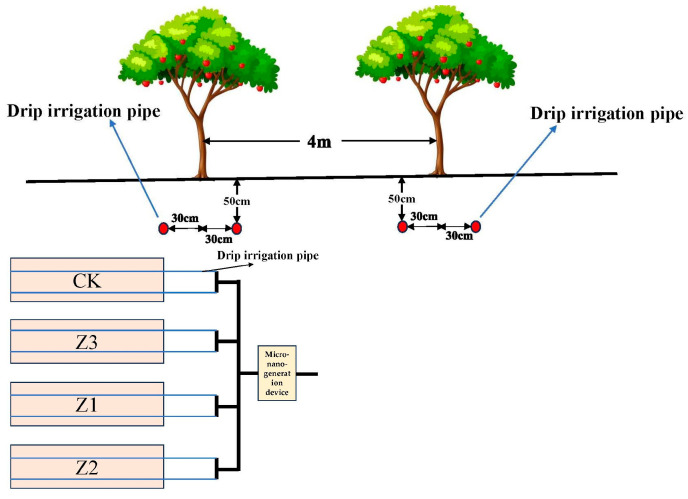
Planting pattern of the experimental field.

Fruit tree variety is red fuji variety New Red 2, rootstock is Malus robusta Rehder, tree shape is free fusiform, tree age 10a (2023), fruit tree plant spacing is 3 m, row spacing is 4 m, prior to the experiment, there was little variability in the growth status of all trees. The experimental design of the different drip irrigation buried depth gradient (Complete Random Design), drip irrigation pipe depth are surface drip irrigation (CK) and underground 10 cm (Z1), 30 cm (Z2) and 50 cm (Z3) drip irrigation, a total of four experimental treatments. Each experimental treatment had 1 row of trees (7 fruit trees, 7 repetitions) and each experimental treatment covered an area of 84 m^2^. We arranged a 16 mm diameter drip irrigation pipe (Figure 2) along the center line of a row of trees 30 cm to the left and 30 cm to the right, with drip holes spaced 50 cm apart and a drip head flow rate of 2 L h^−1^.

We connected a micro-nano-generation device (Irrigation water aerator, China, Summer Spring Technology Corporation) to the irrigation main pipeline common to the four experimental treatments to oxygenate the irrigation water, which could mix the water and air in the irrigation pipeline (increase the gaseous oxygen and dissolved oxygen in the irrigation water) to form micro-nano-bubbly water, and the micro-nano-bubbles in the irrigation water had particle sizes ranging from 200 nm to 4000 nm, with bubble content ranging from 84% to 90%, as a result of our measurements, we determined that the four treatments had the same concentration of micro- and nano-bubbled water and the same amount of irrigation water. Moreover, we installed a water meter at the front of the water inlet of the micro-nano generation device to measure the amount of pure irrigation water (the amount of water without air). The total amount of irrigation water for all treatments in the experiment was 5250 m^3^ ha^−1^, of which 164.1 m^3^ ha^−1^ was irrigated each time from flowering and fruiting stage to young fruit stage, and 273.3 m^3^ ha^−1^ from fruit expansion stage to maturation stage, and the irrigation cycle was determined based on the local drip irrigation cycle for apple orchards, which was set at 7 days, with a total of 24 irrigations in the annual cycle. Water-soluble fertilizers were applied dropwise with each irrigation, and the total amount of N:P_2_O_5_:K_2_O fertilizers applied to apples during the whole life cycle was 150:90:150 kg ha^−1^. We installed a fertilizer tank (shared by the four experimental treatments) at the outlet of the ultra-micro-nano aerator for drip application of water-soluble fertilizer. Pruning, combing, spraying and other management techniques were the same in all treatments.

### 2.3. Measurements of Soil and Plant

(1) Soil water content

Hydra sensors (FDR, Stevens Water Monitoring Systems, Inc., Portland, OR, USA) were buried at six depths in the upper 100 cm of soil (10, 20, 40, 60, 80 and 100 cm); data were recorded at 30 min intervals. Hydra sensors measurement were the volumetric water content of soil, and we used the soil drying method and soil bulk density data to calibrate the data measured by the Hydra sensors instrument to ensure the reliability and accuracy of the soil water content measurement data.

(2) Leaf nitrogen (N), phosphorus (P), and potassium (K) contents

During the fruit expansion stage, 3 groups of leaves were randomly collected from each treatment (across 3 trees), then dried in an oven at 105 °C for 30 min, dried to constant weight at 80 °C, and then crushed and sieved (0.1 mm). Total N was determined using the Kjeldahl method, total P by vanadium-molybdenum yellow colorimetry, and total K by flame photometry.

(3) New shoot tip abscisic acid (ABA), indoleacetic acid (IAA), total gibberellin (GA), and zeatin riboside (ZRs) contents

Five new tips were randomly picked each treatment during the fruit expansion stage, and frozen in liquid N. An appropriate amount of liquid N was then added to 0.5 g samples for grinding. After sample extraction, sample purification, and separation using liquid chromatography, ABA, IAA, GA, and ZRs contents were determined by liquid chromatography-mass spectrometry (LC-MS).

(4) Plant water potential

On a sunny day, three leaves with the same orientation were randomly picked from three sample trees. Approximately 1.5 to 2 h prior to Psi measurements, leaves were placed in aluminized bags to bring them into equilibrium with the xylem potential. Plant water potential was determined using a plant water potential meter (ZWS-I, Beijing Jinyang Wanda Technology Co., Ltd., Beijing, China) between 8:00 and 20:00, with measurements made every 2 h for 3 d during each vegetation season.

(5) Leaf SPAD

Three leaves of the same age as the environment subjected to the light were randomly selected from three branches of three trees, and all leaves were labeled. The same leaf was measured each time, using a chlorophyll meter (SPAD-502, Minolta, Tokyo, Japan).

(6) Photosynthetic data

Typical sunny weather was selected, and a Portable Photosynthesis System (Li-6800, LI-COR Inc., Lincoln, NE, USA) was used to determine the light-response characteristics of leaves between 10:00 and 12:00. The relative humidity of the air in the leaf chamber was 55%, and air temperature was 30 °C; and CO_2_ cylinders provided the air, with a 400 ppm CO_2_ concentration in the leaf chamber. Photosynthetically active radiation flux density was con-trolled at 2000, 1800, 1600, 1400, 1200, 1000, 800, 600, 400, 200, 100, 50, and 0 μmol m^−2^ s^−1^ using an artificial light source, and measured for 120 s at each intensity.

The expression for the right-angle hyperbolic modified model is

(1) $$P_{n}=\alpha \frac{1-\beta I}{1+\gamma I}I-R_{d}$$

(2) $$P_{nmax}=\alpha {\left(\frac{\sqrt{\beta +\gamma }-\sqrt{\beta }}{\gamma }\right)}^{2}-R_{d}$$

(3) $$LSP=\frac{\sqrt{(\beta +\gamma )/\beta }-1}{\gamma }$$

where α is the initial slope, *β* is the photoinhibition coefficient, *γ* is the light saturation coefficient, and *I* is the photosynthetically active radiation intensity (μmol m^−2^ s^−1^).

(7) Yield

At the maturation stage, all fruit was picked from three randomly sampled trees in each treatment. Average fruit weight was determined to calculate the yield of a single tree; yield per hectare was obtained by multiplying the yield of a single tree by the number of trees per hectare.

(8) Fruit quality

Fruit quality indexes were determined from mature apples. Fruit quality was determined from three randomly selected apples per treatment, and all fruit quality measurements were taken from three apples randomly selected from each treatment group. Each apple was measured once for each quality indicator, and each treatment had three sets of repeated data. The fruit individual fresh weight (FW) was measured using an electronic scale with an accuracy of 0.1 g. Fruit longitudinal and transverse diameters were measured by Vernier calipers (0.01 mm). A fruit shape index (FSI) was obtained by dividing the fruit longitudinal diameter by the fruit transverse diameter.

(4) $$FSI=\frac{LD}{TD}$$

where FSI is the fruit shape index, *LD* is the longitudinal diameter (mm), *TD* is the transverse diameter (mm).

Fruit hardness (FH) was determined using a fruit hardness tester (Ed-berg GY-4, Dongguan Yongqi Electronic Equipment Co., Ltd., Dongguan, China); fruit color (L, a, b) was determined by colorimeter (NH310 Portable Colorimeter, Shenzhen San En Shi Technology Co. Ltd., Shenzhen, China); soluble solids (SS) were determined using a Sowei Brix Meter (SW-93T, Sowei Instruments, Guangzhou, China); Determination of titratable acids (TA) using NaOH at a concentration of 0.1 N; the solid–acid ratio (SAR) was obtained from the ratio of soluble solids to titratable acid content; and VC content was determined by spectrophotometry.

### 2.4. Data Processing

Microsoft Excel 2019 was used to organize data; Origin 2021 was used for plotting, data fitting, and principal components analysis (PCA); one-way ANOVA (*p* < 0.05), and the least significant difference (LSD) test were performed in SPSS19.0. GENESCLOUD (https://www.genescloud.cn/home) (accessed on 2 April 2025) is used as a partial mantel correlation analysis and generates a heat map of the correlation network. Partial mantel analysis allows direct analysis of the direct correlation between two sets of variables, controlling for the effect of a third variable on the others, and thus more accurately assess the true correlation between two variables.

## 3. Results

### 3.1. Soil Water Content

**Figure 3:**
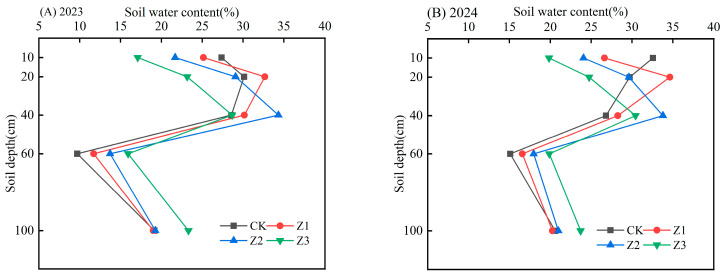
Soil water content during fruit expansion in 2023 (**A**) and 2024 (**B**). Note: CK is surface aerated drip irrigation; Z represents subsurface aerated drip irrigation depths: Z1 (10 cm), Z2 (30 cm), and Z3 (50 cm).

Figure 3 showed the distribution of soil moisture content during the fruit expansion stage of different treatments. Soil water contents at 10 cm in the control (CK) were higher than in treatments; soil evaporation would also be higher in the CK. The highest water contents occurred at 20 cm (Z1 treatment) and 40 cm (Z2 treatment) at the main rooting depth. The highest water contents in treatment Z3 were at 60 and 100 cm.

### 3.2. New Shoot Endogenous Hormone Contents

**Figure 4:**
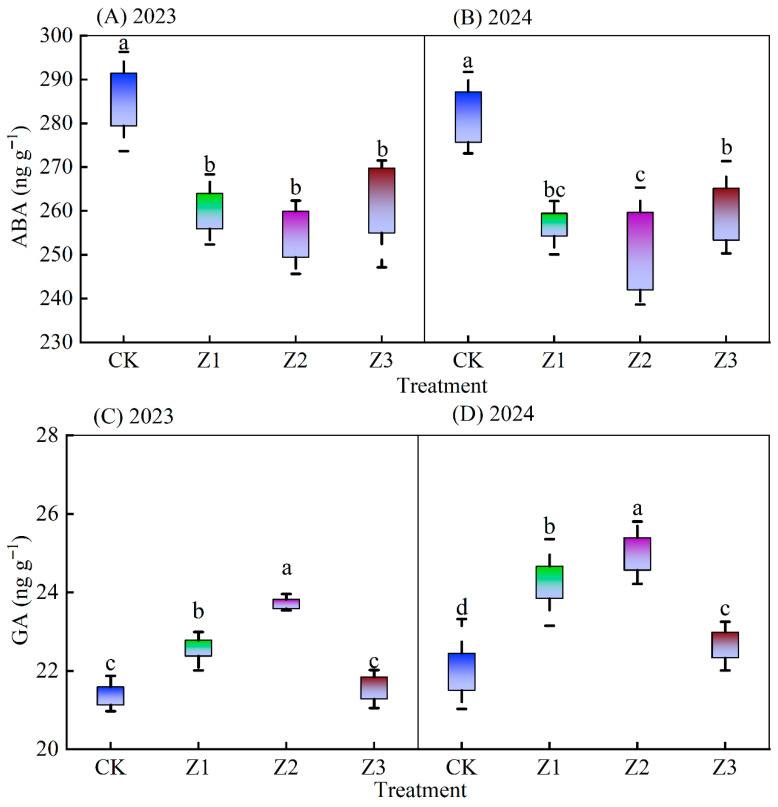

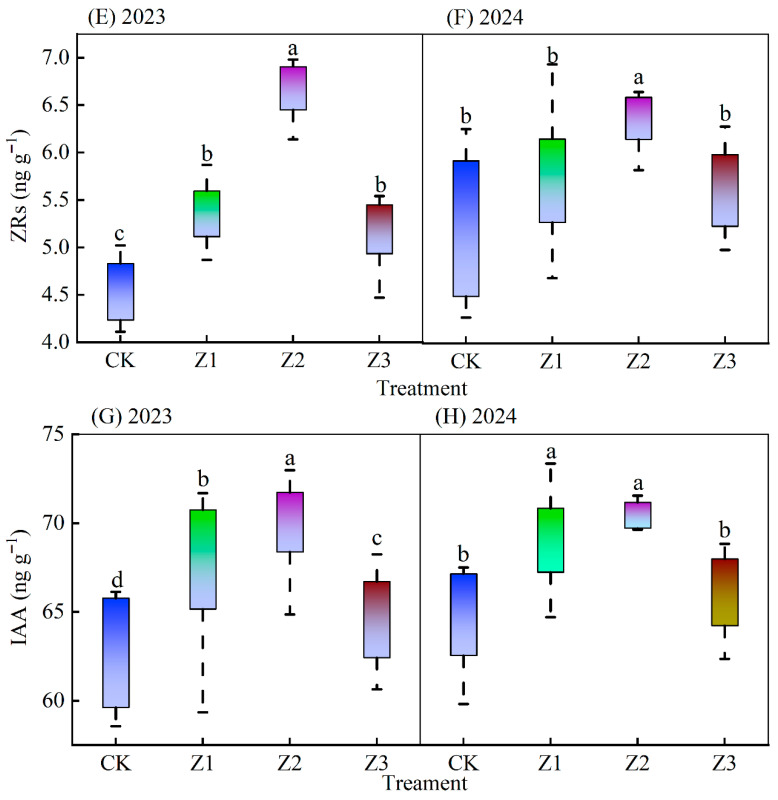
Endogenous hormone content in new tips during 2023 and 2024 growing seasons. Note: Data are means ± standard deviations; different letters above whiskers indicate significant differences (*p* < 0.05). ABA is new shoot tip abscisic acid; IAA is new shoot tip indoleacetic acid; GA is new shoot tip total gibberellin; ZRs is new shoot tip zeatin riboside. CK is surface drip irrigation; Z1 is the drip irrigation pipe buried at 10 cm depth; Z2 is the drip irrigation pipe buried at 30 cm depth; Z3 is the drip irrigation pipe buried at 50 cm depth. 2023 and 2024 refer to the 2023 and 2024 growing seasons in figures, respectively.

ABA content of new stem tips in each vegetation season and treatment trended upwards, then downwards with increased drip irrigation tube burial depth (Figure 4). Compared with the CK, new stem tip ABA contents were reduced on average by 9.06% (Z1), 11.04% (Z2), and 8.18% (Z3) for the two vegetation seasons. Differences in ABA content between the CK and Z1, Z2, Z3treatments were significant (*p* < 0.05). Differences in ABA between the Z1, Z2, and Z3 treatments were not significant. The GA content of Z1 and Z2 treatments in both years was enhanced by an average of 7.92% and 12.30%, respectively, compared with that of CK, and showed significant differences with CK treatment. The IAA content of Z1 and Z2 treatments increased by an average of 7.80% and 11.13%, respectively, compared with the CK treatment, and showed significant differences with CK and Z3. The Z2 treatment had the highest ZRs content among all treatments, with an average enhancement of 37.88% over the CK treatment, which was significantly different from the values of CK, Z1 and Z3 treatments.

### 3.3. Leaf Nutrient Contents

**Figure 5:**
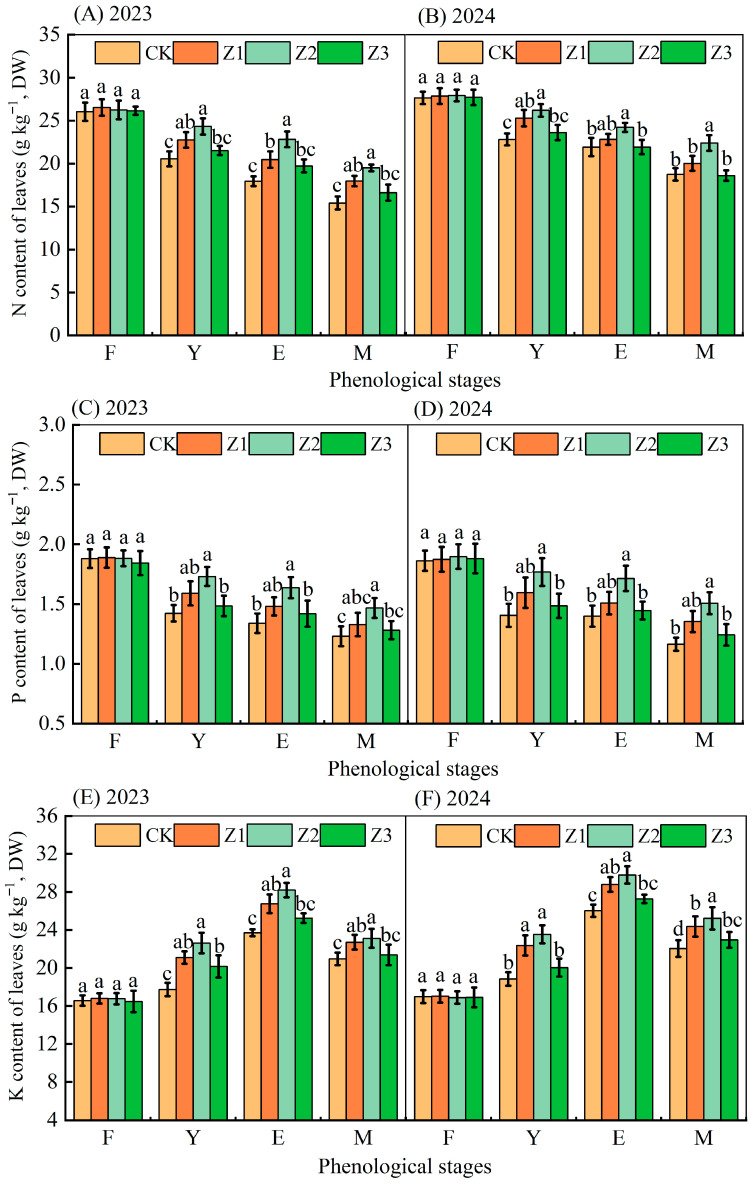
N, P, and K contents of apple leaves at different reproductive stages in 2023 and 2024. (Stage: F, flowering and fruiting; Y, young fruit; E, fruit expansion; M, maturation). Note: Data are means ± standard deviation; different letters above bars indicate significant differences (*p* < 0.05). CK is surface drip irrigation; Z1 is the drip irrigation pipe buried at 10 cm depth; Z2 is the drip irrigation pipe buried at 30 cm depth; Z3 is the drip irrigation pipe buried at 50 cm depth. 2023 and 2024 refer to the 2023 and 2024 growing seasons in figures, respectively.

Characteristics of N, P, and K contents of leaves during vegetation season differed between treatments (Figure 5). Leaf N and P content of different treatments gradually decreased with the advancement of fruit development, and leaf K content showed a pattern of first increasing and then decreasing with the advancement of fruit development. N, P, and K contents of leaves in the four treatments did not differ between treatments both years at the flowering and fruiting stage (*p* > 0.05), but they did differ significantly during flowering and fruit set stage, fruit expansion stage, and maturation stage (*p* < 0.05). For both years, taking the fruit expansion stage as an example, compared with CK, mean leaf N contents increased in treatments Z1, Z2, and Z3 by 8.63%, 18.03%, and 4.51%, respectively; mean P contents increased by 9.21%, 22.42%, and 4.69%, respectively; and mean K contents increased by 11.68%, 16.63% and 5.62%, respectively, while treatments Z1 and Z2 enhanced leaf N, P, and K contents.

### 3.4. Plant Water Potential

**Figure 6:**
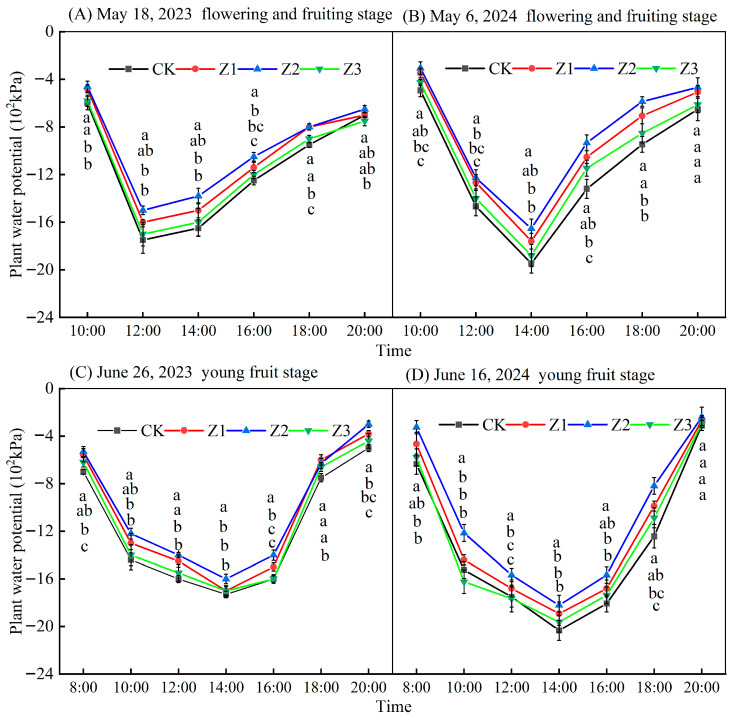

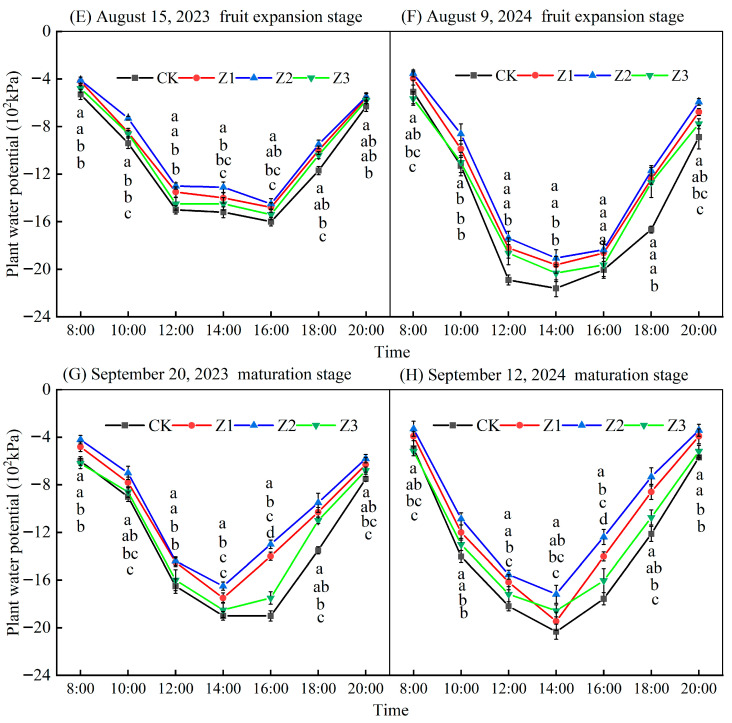
Daily changes in plant water potential by different reproductive stages, 2023 and 2024. Note: Data represent means ± standard deviation; different letters above symbols indicate significant differences (*p* < 0.05). CK is surface drip irrigation; Z1 is the drip irrigation pipe buried at 10 cm depth; Z2 is the drip irrigation pipe buried at 30 cm depth; Z3 is the drip irrigation pipe buried at 50 cm depth.

Figure 6 showed the daily variation in plant water potential at different dates in 2023 and 2024. Daily changes in plant water potential for both years initially trended down, then increased with time. The overall plant water potential in treatments was Z2 > Z1 > Z3 > CK. Compared with CK values (May, 2023 and 2024), plant water potential in Z1, Z2, and Z3 treatments increased by 11.88%, 17.40%, and 4.16%, respectively; in June (2023 and 2024) this was 8.95%, 15.21%, and 3.25%, respectively; in August (2023 and 2024) this was 11.32%, 15.58%, and 6.65%; for September (2023 and 2024) these values were 15.17%, 21.46% and 6.40%, respectively. Treatment Z2 had the highest average daily plant water potential and treatment CK the lowest.

### 3.5. Apple Leaf SPAD and Light Response Characteristics

#### 3.5.1. Leaf SPAD Values During the Reproductive Period

**Figure 7:**
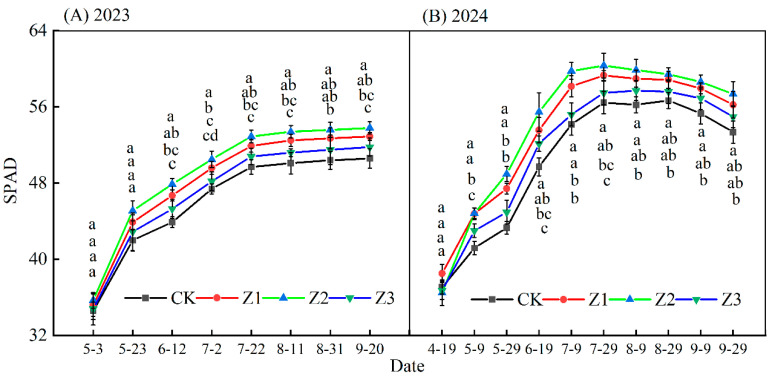
Leaf SPAD values by different reproductive stages, 2023 and 2024. Note: Data are means ± standard deviation; different letters above symbols indicate significant differences (*p* < 0.05). CK is surface drip irrigation; Z1 is the drip irrigation pipe buried at 10 cm depth; Z2 is the drip irrigation pipe buried at 30 cm depth; Z3 is the drip irrigation pipe buried at 50 cm depth.

In 2023, as apple reproductive stages, leaf SPAD values in treatments initially trended upwards, then stabilized (Figure 7); for 2024 these values initially trended upwards, then downwards. Different drip irrigation depths significantly affected leaf SPAD values in both years (*p* < 0.05). For both years, 3 May and 19 April, there were no significant differences in leaf SPAD values between treatments (*p* > 0.05), with SPAD values ranked Z2 > Z1 > Z3 > CK. Compared with CK values, during the fruit expansion stage in both years (11 August (2023) and 9 (2024)), leaf SPAD values were increased by 4.47% (Z1), 6.06% (Z2), and 2.24% (Z3), respectively. Compared with CK values, during the maturation stage in both years (20 September (2023) and 29 (2024)), leaf SPAD was increased by 4.56% (Z1), 6.36% (Z2), and 2.47% (Z3).

#### 3.5.2. Leaf Light Response During the Fruit-Expansion Stage

**Figure 8:**
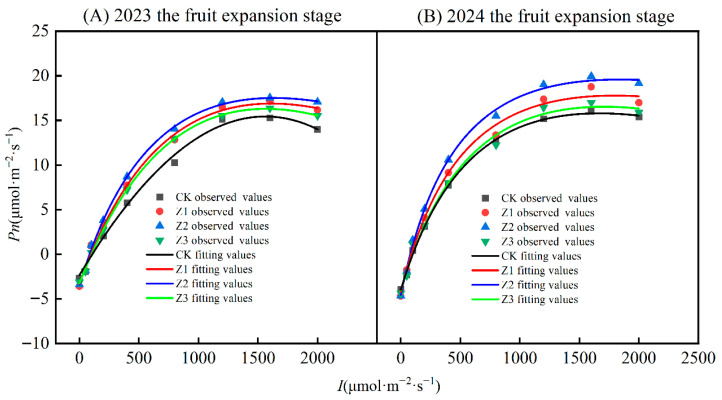
Fitted light response of apple leaves in 2023 and 2024. Note: *Pn* is the net photosynthetic rate of the leaf. *I* is photosynthetically active radiation. CK is surface drip irrigation; Z1 is the drip irrigation pipe buried at 10 cm depth; Z2 is the drip irrigation pipe buried at 30 cm depth; Z3 is the drip irrigation pipe buried at 50 cm depth.

Light-response characteristics in treatments for both years showed the same pattern (Figure 8 and Table 1). *α* values for each treatment ranked Z2 > Z1 > Z3 > CK. Compared with the CK, *β* values reduced by 31.43% (Z1), 41.02% (Z2), and 21.43% (Z3) in both growing seasons. In both growing seasons, *γ* values in treatment Z2 were greatest, and in treatment CK lowest. Dark respiration rate (*R_d_*) indexes followed the same pattern. Compared with the CK, the *Pn_max_* value for Z1, Z2, and Z3 treatments increased by 11.12%, 18.91%, and 5.19%, respectively. In both years, the light-saturation point in Z2 treatment was greatest and that in CK treatment lowest; the light compensation and light saturation points in different treatments showed opposite patterns. We define the difference between light saturation and light compensation points as a range of plant photosynthesis in response to light intensity. For 2023, light flux utilization ranges in the CK and Z1, Z2, and Z3 treatments were 1455.83, 1518.27, 1547.98, and 1465.20 μmol m^−2^ s^−1^; for 2024 these values were 1566.69, 1709.22, 1756.93, and 1606.86 μmol m^−2^ s^−1^, respectively. In both years, treatment Z2 had the largest range of light flux utilization and the CK treatment the lowest.

### 3.6. Apple Yield, Quality, and Correlation Analysis

#### 3.6.1. Apple Yield and Quality

Table 2 was an indicator of apple yield. Single fruit weight, fruit shape index, fruit hardness, L (The lightness and darkness of colors), a (Redness and greenness of colors), VC content, and yield of different treatments trended upwards, then decreased with increased drip irrigation burial depth. b showed a gradual decrease in trend in 2023, and showed a gradual increase in the change rule in 2024. It showed that L and b were not affected by the depth of drip irrigation. In both years, only single fruit weight of Z2 treatment showed a significant difference with all other treatments and was 16.68% higher than CK treatment. Fruit shape index was not significantly different for all treatments. In both vegetation seasons, only the Z2 treatment showed significant differences with CK for L. The c comparisons of the Z2 treatment with the CK treatment showed significant differences only in 2024. In both growing seasons, soluble solids, titratable acid, sugar to acid ratio, and VC of Z2 treatment showed significant difference from CK treatment. In both vegetation season, the yield was shown to increase with the increase in the buried depth of the drip irrigation pipe and then decrease the change rule, and the yield of Z2 treatment was the highest among all treatments, and the yield of the two years compared with that of CK was increased by 8.72% on average.

#### 3.6.2. Correlation Analysis of Apple Yield and Quality

**Figure 9:**
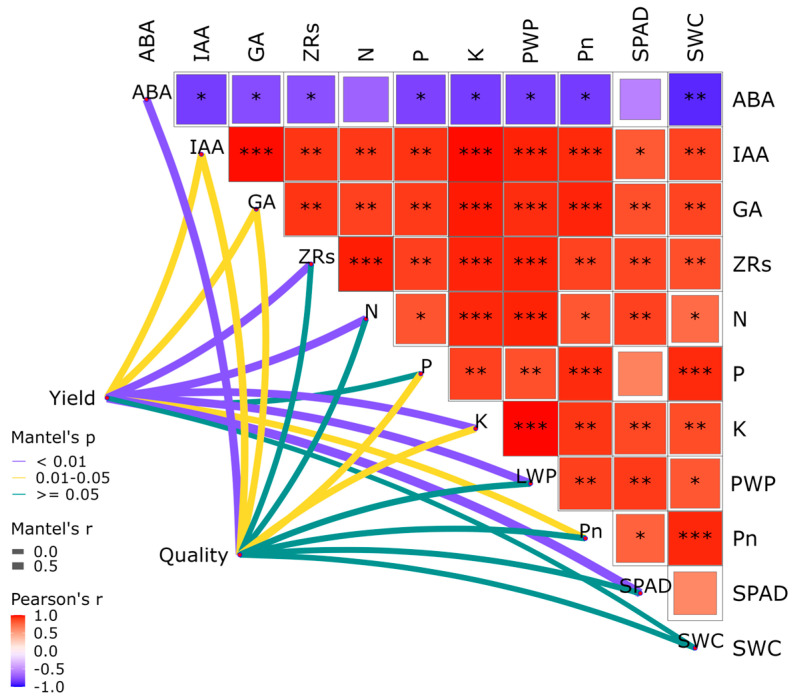
Analysis of key factors affecting yield and quality in apples in 2023 and 2024. Note: ABA is the abscisic acid content in the tip of a new shoot; IAA is the indole acetic acid content in the tip of a new shoot; GA is the total amount of gibberellins in the tip of a new shoot; ZRs is the zeatin riboside content in the tip of a new shoot; N is the nitrogen content of leaves during fruit expansion; P is the phosphorus content of leaves during fruit expansion; K is Leaf potassium content of leaves during fruit expansion; LWP is the average daily plant water potential during fruit expansion; Pn is the potential maximum net photosynthetic rate during fruit expansion; SPAD is the relative chlorophyll content of leaves during fruit expansion, and SWC is soil moisture during fruit expansion. * Denotes 0.01 < *p* ≤ 0.05; ** Denotes 0.001 < *p* ≤ 0.01; *** Denotes *p* ≤ 0.001.

Partial mantel analysis was performed between yield and overall quality (L, a, b, SS, TA, SA, VC, WF, and FSI) of apples in 2023 and 2024 with physiological indicators (Pn, SPAD, PWP, ZRs, ABA, GA, IAA, N, P, and K) and soil moisture content, as well as Pearson analysis between physiological indicators and soil moisture content (Figure 9). The highly significant correlations (*p* < 0.01) with yield were ZRs, leaf N content, leaf K content, PWP and SPAD, and the significant correlations (*p* < 0.05) with yield were IAA, GA, and Pn. All the correlations were significant with Pn, and all the correlations were significant with SPAD except for ABA and leaf P content. The highly significant correlation with the overall quality of apple was ABA, and the significant correlation with the overall quality of apple was IAA, GA, leaf P content and K content. Therefore, the quality of apples can be improved by regulating leaf N content, K content, and endogenous hormone content.

### 3.7. Principal Component Analysis of Yield and Quality Indicators

#### 3.7.1. Correlation Between Yield and Quality Indicators

**Figure 10:**
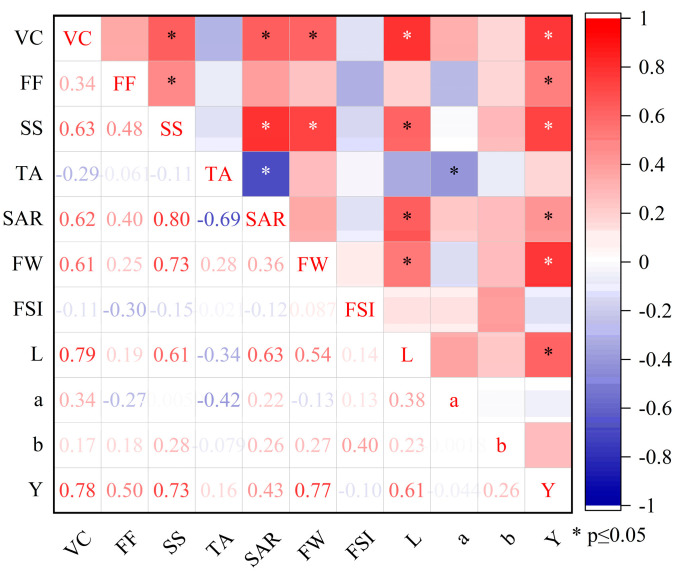
Correlation analysis of yield and quality indicators in 2023 and 2024. Note: VC is vitamin C; FF is fruit firmness; SS is soluble solids; TA is titratable acid; SAR is sugar to acid ratio; FW is the fruit individual fresh weight; FSI is fruit shape index; L is lightness and darkness of colors; a is redness and greenness of colors; b is yellowness and blueness; Y is apple yield.

Figure 10 showed the correlation analysis of yield and quality indicators for 2023 and 2024. VC was significantly and positively correlated with SS, SAR, FW, and color difference (L) with yield. Yield showed correlation with color difference (L), FW, SAR, SS, FF and VC. FSI and color difference b had no correlation with other indicators, and a had correlation with TA. Therefore, FSI and b were excluded when performing principal component analysis.

#### 3.7.2. Evaluating the Effects of Subsurface Oxygenated Drip Irrigation on Yield and Quality Indicators

**Figure 11:**
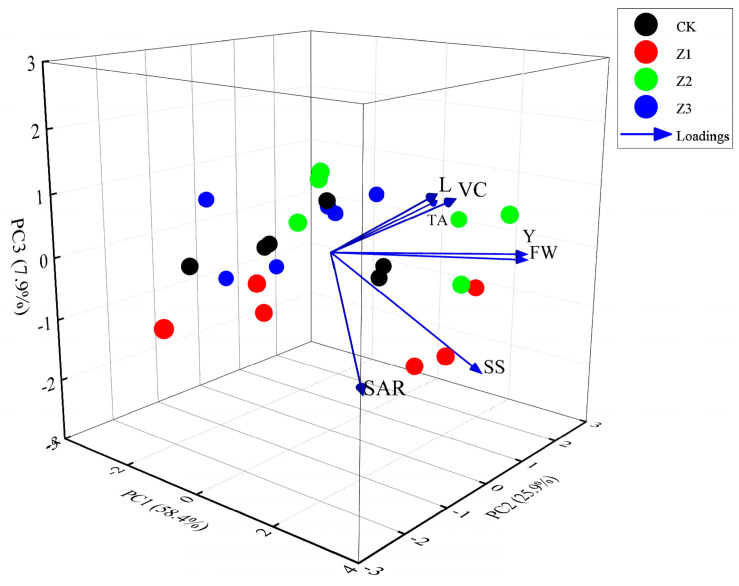
Principal component analysis of apple yield and quality indexes by treatment for 2023 and 2024. Note: VC is vitamin C; FF is fruit firmness; SS is soluble solids; TA is titratable acid; SAR is sugar to acid ratio; FW is the fruit individual fresh weight; FSI is fruit shape index; L is lightness and darkness of colors; a is redness and greenness of colors; Y is apple yield. CK is surface drip irrigation; Z1 is the drip irrigation pipe buried at 10 cm depth; Z2 is the drip irrigation pipe buried at 30 cm depth; Z3 is the drip irrigation pipe buried at 50 cm depth.

Because of correlations between yield and quality indexes, we evaluated fruit yield and quality indexes by downgrading indexes and simplifying index factors using factor analysis in 2023 and 2024 (Figure 11). The first principal component (PC1) of contained 58.4% of all original information, and its size was mainly affected by VC, SS, SAR, FW, L and Y. PC2 contained 25.9% of original information, and its size was mainly affected by TA, SAR, FW and Y. PC3 contained 7.9% of original information, and was mainly affected by VC, SS, SAR and L. Based on these results, expressions of three principal components and one composite score were obtained:

F1=0.43VC+0.45SS−0.12TA+0.39SAR+0.39FW+0.41L+0.36Y

F2=−0.07VC+0.05SS+0.71TA−0.39SAR+0.37FW−0.13L+0.42Y

F3=0.49VC−0.54SS+0.01TA−0.42SAR−0.05FW+0.53L−0.04Y

CS=0.63F1+0.28F2+0.09F3

Through these formulas, for 2023 and 2024, treatment Z2 had both the best apple yield and quality, and treatment Z2 had the highest composite score.

## 4. Discussion

(1) Effect of subsurface oxygenated drip irrigation on soil water and oxygen distribution

Subsurface drip irrigation maintains relatively dry soil surface and good moisture conditions in the root zone, reduces surface water loss, promotes root growth, and improves water and nutrient uptake and utilization [19]. The O_2_ content in the soil was significantly higher than that of ordinary subsurface drip irrigation after oxygenated irrigation, which delayed the crowding out of soil oxygen by drip irrigation [20]. Combined with the two, the microbubbles in the two-phase flow of water and gas in oxygenated irrigation were easily attached to the soil pore space, which could supply oxygen to the water in a continuous manner and maintain a good oxygen environment in the soil [21]. In actual soil, the non-moist soil may also be anoxic [22]; however, aerobic irrigation not only has the effect of increasing oxygen in moist soil, but also has a certain effect on the oxygen content of non-moist soil, and the aerobic water will drive the microbubbles to continuously diffuse around the soil, which will increase the oxygen content in non-moist soil [23].

Currently, there is a controversy about the effect of buried depth of subsurface drip irrigation pipes on crop yield increase, some studies have shown that increasing depth of drip irrigation pipe burial stabilized after increasing crop yield [20], the reason is that the increased depth of drip irrigation increases the diffusion paths of gas movement, the distribution of soil moisture is more uniform, and the range of wetted soil is larger [24], it also promotes the growth and distribution of crop roots [25], and facilitates water uptake by crop roots. Studies by some other experts have shown that an increase in drip depth reduces crop yields [26] because with a gradual increase in drip depth, the resistance to shallow water increases, which gradually reduces the water content of the surface soil [27], which is detrimental to the uptake of water and nutrients by the surface root system of the crop. The main point of contention is in the different effects of burial depth of drip irrigation pipes on the distribution of soil moisture content. In our study, the surface soil moisture content gradually decreased, and the deep soil moisture content gradually increased with increasing depth of drip irrigation pipe burial. The rootstock of our apple trees was Malus robusta Rehder, and the root system of this rootstock adapts to the moisture depth of the irrigation system, exhibiting a vertical distribution mainly concentrated between 0 and 60 cm [28], and among all the treatments, only the drip irrigation pipe of the Z2 treatment was in the middle of the main root layer of apples, thus facilitating the uptake of water and fertilizers by the root system of apples.

(2) The effect of subsurface oxygenated drip irrigation on hormone and nutrient uptake in apples

Plant endogenous hormones are a class of trace organic substances produced by the plant’s own metabolism, which have obvious physiological effects at very low concentrations, and can play a role in regulating the physiological effects of plant growth and differentiation, flowering and fruiting, ripening and senescence, and dormancy and abscission [29,30]. Among them, IAA, GA3, and ZRs are usually considered as growth-promoting hormones, which have the effect of promoting plant growth and fruit growth [31]. ABA is a growth inhibitor, which has the ability to coordinate the metabolism in the body of the crop, but its high level can lead to accelerated organ senescence [32] and is unfavorable to crop yield [33]. Hormones have the same regulatory effects on fruit trees, and they mainly affect the growth process of fruit trees, in which the tree size and branch growth of fruit trees are mainly directly related to IAA and GA, and GA can play a role in elongating the length of fruit stalks and improving the fruit shape index [34]. In our study, the contents of IAA, GA, and ZRs in new shoot tips were increased and the contents of ABA were decreased by subsurface oxygenated drip irrigation compared with surface oxygenated drip irrigation, indicating that subsurface oxygenated drip irrigation is beneficial to promote the growth and yield of apples. Correlation analysis showed that IAA, GA, ZRs, and ABA were not significantly correlated with apple yield, but they were significantly correlated with Pn, indicating that these hormones play an indirect role in apple yield (Figure 9). In our study, the levels of IAA, GA and ZRs showed a pattern of increase and then decrease with the buried depth of drip irrigation pipe, and ABA showed the opposite change rule (Figure 4), and the effect of oxygen-enhanced drip irrigation with the buried depth of drip irrigation pipe of 30 cm was the most obvious effect on the promotional hormones, which was mainly related to the buried depth of drip irrigation pipe in the position of the apple rhizosphere, and the root system was more sensitive to the soil water, nutrients and oxygen, and the roots quickly sensed the stress to synthesize chemical signals to produce the hormones. The root system is sensitive to soil water and oxygen, and when stress occurs, the root system rapidly senses it, transmits it to the aboveground in the form of synthesized chemical signals, and regulates the aboveground physiological functions of the crop through the synthesis of related hormones [35], especially when the root system is hypoxic, it produces abscisic acid and limits the transport of water and minerals between the roots and stems [36].

The level of nutrient supply affects physiological processes such as the synthesis and metabolism of endogenous hormones in plants [37], where a decrease in phosphorus leads to a decrease in cytokinin [38], and sufficient nitrogen and potassium promote the growth of root meristematic tissues and aboveground, the synthesis of cytokinin, and the accelerated catabolism of ABA content [39]. Some studies have shown that endogenous hormones also increase plant nutrient accumulation [40], so endogenous hormone content and plant nutrient accumulation are mutually reinforcing. Subsurface oxygenated drip irrigation promotes plant nutrient uptake and accumulation by increasing soil enzyme activity and microbial activity and decomposing organic matter, thereby promoting the release and decomposition of mineral elements in the soil [41]. In our study, there was no significant difference in leaf N, P, and K contents between subsurface oxygenated drip irrigation and the control treatment at the flowering and fruiting stage because the flowering and fruiting stage was a relatively short period of time before the experiment was carried out, and thus the treatments did not show significant differences among each other. The leaf N, P, and K contents of the other three fertility stages were differently enhanced by subsurface oxygenated drip irrigation compared with the control treatment (Figure 5).

(3) Effects of subsurface oxygenated drip irrigation on SPAD, leaf photosynthetic characteristics and plant water potential in apple

Oxygen deficiency in the crop root system causes crop stomata to close and plant water potential to decrease, which affects the normal physiological function of the crop [42] and hinders high crop yield. Chlorophyll, as the main pigment in leaf chloroplasts, can reflect the strength of leaf photosynthetic capacity and plant growth, and its content can be expressed by leaf SPAD value [43]. Leaf photosynthetic capacity is the basis of crop growth and development and crop yield [44]. The main factors affecting plant photosynthesis are biochemical limitation, stomatal limitation and chloroplastic limitation [45]. In terms of biochemical limitation, it is mainly related to photosynthetic enzyme activities, especially Rubisco enzyme activities [46]. In terms of stomatal limitation, when the microenvironment of the crop root system changes, the root system rapidly senses it and regulates the aboveground physiological functions of the crop through endogenous hormones, e.g., reduction in leaf stomatal conductance and photosynthesis rate, and slowing down of the growth of the crop leaves [47,48]. In terms of chloroplastic limitation, it is mainly related to the chlorophyll content of leaves [49]. In our study, the enhancement effect of subsurface oxygenated drip irrigation on leaf photosynthesis was mainly in three aspects, first, subsurface oxygenated drip irrigation increased the aerobic respiration efficiency and activity of the root system [50,51], which facilitated the uptake of nitrogen by the root system and its translocation to the aboveground part [52], contributing to the leaf nitrogen content (Figure 5), and enhanced Rubisco enzyme activity. Second, subsurface oxygenated drip irrigation increased leaf chlorophyll content (Figure 7), enhanced the range of light energy absorption (Table 1), and promoted the stomatal conductance and transpiration rate of the crop [53], which in turn improved the photosynthetic performance and plant water potential of the aboveground plant leaves (Figure 6 and Figure 8). Third, subsurface oxygenated drip irrigation reduced the ABA content in the stem tips of the new shoots (Figure 4), increased leaf stomatal conductance, and promoted leaf photosynthesis and plant water potential.

(4) Pathway analysis of subsurface oxygenated drip irrigation on apple yield increase and quality improvement

Soil fertility is the most important factor affecting the yield and quality of fruit trees in the absence of growth stress [54]. In our study, we used the partial mantel method to analyze the correlation between yield and quality of apple and physiological indicators and soil moisture content. This method can directly analyze the direct correlation between yield and quality and other indicators, eliminating the influence of indirect factors. The results of this study showed that apple yield showed highly significant correlation with SPAD, leaf N and K content, and only significant correlation with Pn and SWC, mainly because it is the products of crop photosynthesis is not only used to enhance the crop yield, but also used for the crop’s own nutrient growth, so it showed significant correlation (Figure 9). The significant correlation between SWC and yield indicated that there was a certain amount of water stress in our four experimental treatments. Apple quality had highly significant correlations with ABA, significant correlations with LWP, ZRs, IAA, SWC, and leaf N and K contents, but no significant correlations with Pn. The main reason may be that we pooled all the quality indexes (L, a, b, SS, TA, SA, VC, WF, and FSI) into one comprehensive quality index, thus reducing the correlation with Pn correlation (Figure 9). Considering the significant correlations with apple yield and quality indicators, as well as the correlations between physiological indicators, subsurface oxygen drip irrigation mainly affects apple photosynthesis by regulating endogenous hormone content and enhancing leaf mineral content, which ultimately increases apple yield and improves the quality of apples.

## 5. Conclusions

Subsurface aerobic drip irrigation reduced surface soil water content, increased soil water content in the apple rhizosphere, improves plant water potential, increased new tip gibberellin, indoleacetic acid and zeatin riboside content, and reduced new tip abscisic acid content. Leaf nitrogen, phosphorus, and potassium contents were elevated during young fruit, fruit expansion, and maturation stages, and leaf SPAD was increased, increasing the range of light energy utilization of the leaves and promoting the potential maximum net photosynthetic rate. Significant correlations between IAA, GA, ZRs, leaf N content, leaf K content, LWP, Pn, SPAD, and apple yield were determined by partial mantel analysis. A significant correlation was found between ABA, IAA, GA, leaf P, and K content and apple quality. Therefore, the quality of apples can be improved by regulating leaf N content, K con-tent and endogenous hormone content. Fruit quality indexes and yield indexes of different treatments had different performances, treatment Z2 had both the best apple yield and quality, and through constructing the principal component analysis of yield and quality indexes for 2 years, we obtained the highest comprehensive score of underground 30 cm oxygenated drip irrigation treatment.

## Figures and Tables

**Table 1:** Eigenvalues of nonrectangular hyperbolic modified model fit by treatment and year.

Growing Season	Treatment	*α*	*β*	*γ*	*R_d_*	*Pn_max_*	*I_sat_*	*I_c_*
2023	CK	0.0227	0.00032	0.00002	2.35 ± 0.14	15.41 ± 0.78	1563.20 ± 112.36	107.37 ± 4.59
	Z1	0.0375	0.00021	0.00062	3.30 ± 0.21	16.89 ± 0.52	1613.12 ± 129.24	94.85 ± 2.78
	Z2	0.0444	0.00018	0.00089	3.45 ± 0.18	17.52 ± 0.61	1632.86 ± 79.67	84.88 ± 3.49
	Z3	0.0347	0.00023	0.0005	3.26 ± 0.22	16.29 ± 0.73	1566.17 ± 147.55	100.97 ± 3.66
2024	CK	0.0442	0.00017	0.00103	3.97 ± 0.11	15.79 ± 0.37	1667.58 ± 124.56	100.89 ± 6.42
	Z1	0.0541	0.000126	0.0013	4.21 ± 0.18	17.78 ± 0.76	1797.30 ± 99.24	88.08 ± 3.79
	Z2	0.0653	0.000109	0.00161	4.57 ± 0.24	19.58 ± 0.45	1836.62 ± 86.58	79.69 ± 4.25
	Z3	0.0453	0.000155	0.00103	4.04 ± 0.23	16.53 ± 0.71	1706.67 ± 134.79	99.81 ± 3.97

Note: Data are means ± standard deviations. *α*, *β*, *γ*, *R_d_*, *Pn_max_*, *I_sat_*, and *I_c_* represent initial slope, photoinhibition coefficient, light saturation coefficient, dark respiration rate, maximum net photosynthetic rate, light saturation intensity, and light compensation point, respectively. The larger the initial slope, the higher the efficiency of light-energy utilization at lower light intensities. A larger photoinhibition coefficient indicates that a plant is more susceptible to photoinhibition and reduced photosynthetic capacity. The light-saturation coefficient is related to the light-saturation point (the light intensity at which the photosynthetic rate of a plant peaks, indicating a plant’s ability to adapt to light). The dark respiration rate is the amount of oxygen consumed and carbon dioxide produced from organic matter by respiration per unit of time and per unit of leaf area when light intensity is zero. The lower the light compensation point, the more shade tolerant a plant is. Below the light compensation point, plant respiration exceeds photosynthesis, and rather than accumulating organic matter, it is consumed.

**Table 2:** Apple yield and quality by treatment and year.

Growing Season	Treatment	FW (g)	FSI	FF (kg cm^−2^)	L	a	b	SS (Brix)	TA (%)	SAR	VC (mg 100 g^−1^)	Yield (kg ha^−1^)
2023	CK	220.33 ± 7.51 b	0.80 ± 0.05 a	6.29 ± 0.25 a	47.55 ± 0.93 d	24.63 ± 2.71 b	12.57 ± 1.73 b	12.13 ± 0.60 b	0.78 ± 0.02 a	15.65 ± 0.50c	1.06 ± 0.12 c	45,029.86 ± 755.28 b
	Z1	231.80 ± 8.66 ab	0.89 ± 0.07 a	6.89 ± 0.71 a	53.60 ± 1.59 b	26.51 ± 2.44 ab	11.67 ± 1.62 ab	13.50 ± 0.12 a	0.66 ± 0.04 b	20.43 ± 1.25a	1.48 ± 0.10 ab	47,044.69 ± 866.44 ab
	Z2	253.03 ± 17.00 a	0.91 ± 0.03 a	6.99 ± 0.54 a	57.57 ± 1.07 a	29.62 ± 2.34 a	10.73 ± 1.18 a	12.93 ± 0.10 a	0.71 ± 0.02 ab	18.17 ± 0.66b	1.55 ± 0.07 a	49,100.29 ± 1299.62 a
	Z3	231.03 ± 8.92 b	0.82 ± 0.08 a	6.55 ± 0.36 a	50.90 ± 1.43 c	26.53 ± 1.50 ab	9.36 ± 0.71 ab	12.87 ± 0.25 a	0.74 ± 0.04 a	17.48 ± 0.75b	1.33 ± 0.05 b	45,688.78 ± 1211.51 b
2024	CK	248.06 ± 10.60 b	0.80 ± 0.06 a	6.89 ± 0.28 a	50.43 ± 1.64 b	22.97 ± 1.93 a	9.01 ± 0.84 b	12.67 ± 0.32 c	0.80 ± 0.02 a	15.83 ± 0.15c	1.38 ± 0.05 b	51,873.62 ± 952.51 c
	Z1	286.18 ± 13.52 a	0.84 ± 0.03 a	7.22 ± 0.30 a	55.58 ± 1.32 a	24.40 ± 3.50 a	11.51 ± 1.28 ab	15.40 ± 0.44 ab	0.74 ± 0.02 b	20.83 ± 1.08ab	1.55 ± 0.03 ab	54,654.25 ± 1321.74 ab
	Z2	291.60 ± 19.91 a	0.87 ± 0.01 a	7.25 ± 0.17 a	57.36 ± 3.44 a	26.70 ± 1.76 a	13.49 ± 1.34 a	14.57 ± 0.59 a	0.76 ± 0.02 b	19.19 ± 1.25 a	1.70 ± 0.17 a	56,257.36 ± 1214.07 a
	Z3	254.25 ± 12.71 b	0.84 ± 0.09 a	7.16 ± 0.36 a	53.99 ± 2.31 ab	23.90 ± 3.11 a	10.39 ± 3.00 ab	13.70 ± 0.62 b	0.77 ± 0.01 ab	17.87 ± 0.79 b	1.41 ± 0.05 b	52,569.26 ± 853.95 bc

Note: Data in the table represent means ± standard deviations; different letters within a column indicate significant differences (*p* < 0.05). FW is the fruit individual fresh weight; FSI is fruit shape index; FF is fruit firmness; L is lightness and darkness of colors; a is redness and greenness of colors; b is yellowness and blueness; SS is soluble solids; TA is titratable acid; SAR is sugar to acid ratio; VC is vitamin C. CK is surface drip irrigation; Z1 is the drip irrigation pipe buried at 10 cm depth; Z2 is the drip irrigation pipe buried at 30 cm depth; Z3 is the drip irrigation pipe buried at 50 cm depth.

## Data Availability

The original contributions presented in this study are included in the article. Further inquiries can be directed to the corresponding authors.
